# CCL19 and CCR7 Expression, Signaling Pathways, and Adjuvant Functions in Viral Infection and Prevention

**DOI:** 10.3389/fcell.2019.00212

**Published:** 2019-10-01

**Authors:** Yan Yan, Renfang Chen, Xu Wang, Kai Hu, Lihua Huang, Mengji Lu, Qinxue Hu

**Affiliations:** ^1^Center of Clinical Laboratory, The Fifth People’s Hospital of Wuxi, Affiliated Hospital of Jiangnan University, Wuxi, China; ^2^The International Joint Research Laboratory for Infection and Immunity (China-Germany), Jiangnan University, Wuxi, China; ^3^Hepatology Institute of Wuxi, The Fifth People’s Hospital of Wuxi, Affiliated Hospital of Jiangnan University, Wuxi, China; ^4^State Key Laboratory of Virology, Wuhan Institute of Virology, Chinese Academy of Sciences, Wuhan, China; ^5^Institute of Virology, University Hospital of Essen, University Duisburg-Essen, Essen, Germany; ^6^Institute for Infection and Immunity, St. George’s, University of London, London, United Kingdom

**Keywords:** CCL19, CCR7, chemotaxis, antivirus, signaling, adjuvant

## Abstract

Chemokine (C–C motif) ligand 19 (CCL19) is a critical regulator of the induction of T cell activation, immune tolerance, and inflammatory responses during continuous immune surveillance, homeostasis, and development. Migration of CC-chemokine receptor 7 (CCR7)-expressing cells to secondary lymphoid organs is a crucial step in the onset of adaptive immunity, which is initiated by a complex interaction between CCR7 and its cognate ligands. Recent advances in knowledge regarding the response of the CCL19-CCR7 axis to viral infections have elucidated the complex network of interplay among the invading virus, target cells and host immune responses. Viruses use various strategies to evade or delay the cytokine response, gaining additional time to replicate in the host. In this review, we summarize the impacts of CCL19 and CCR7 expression on the regulation of viral pathogenesis with an emphasis on the corresponding signaling pathways and adjuvant mechanisms. We present and discuss the expression, signaling adaptor proteins and effects of CCL19 and CCR7 as these molecules differentially impact different viral infections and viral life cycles in host homeostatic strategies. The underlying mechanisms discussed in this review may assist in the design of novel agents to modulate chemokine activity for viral prevention.

## Introduction

CCL19 and its receptor CCR7 control a diverse array of migratory events in adaptive immunity following antigen encounter by immunocytes. Recently, some rules governing the mechanisms of the CCL19-CCR7 axis in directing lymphocyte homing and encountering viruses have been clarified *in vitro* (Comerford et al., 2006; de Paz et al., 2007; Jafarnejad et al., 2017). *In vivo*, both the host’s antiviral and adjuvant-based immune responses are regulated by interactions among viral proteins, chemokine receptors and their downstream adaptor components. Changes in the bioavailability of CCR7/ligands (CCL19 and CCL21) may modulate the immunopathogenesis pathways of the host, thereby altering virus invasion (Comerford et al., 2013; Steen et al., 2014). Hence, we systematically evaluated the contributions of CCL19 and CCR7 expression polymorphisms, signal transduction and CCL19-based adjuvant mechanisms in viral infections.

**FIGURE 1:**
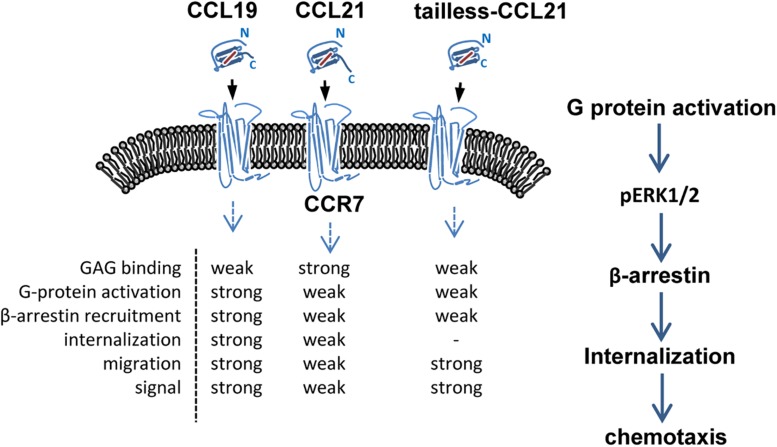
Schematic of CCR7 and its ligands. CCL19, CCL21 and tailless CCL21 bind CCR7, a 7-transmembrane receptor. Binding of receptor/ligands results in GPCR activation and consequent internalization, followed by a decrease in the surface-exposed receptor and activation of certain intracellular pathways. GAG, glycosaminoglycan.

CCL19 and CCL21 have a conserved tetra-cysteine motif but only share 32% amino acid identity. Structurally, CCL21 differs from CCL19 because it has a uniquely long C-terminal tail containing an extra 37 amino acids (6 cysteine residues) that are highly positively charged and capable of binding glycosaminoglycans (GAGs) (Nagira et al., 1997; Steen et al., 2014). CCL19 is secreted by mature dendritic cells (mDCs), while CCL21 is secreted from the endothelium of afferent lymphatic vessels (this has been shown in mice, but evidence in humans is lacking), and both are predominantly secreted by the lumen of high endothelial venules, the stromal cells of the draining lymph node and the spleen (Steen et al., 2014; Wang et al., 2018). Due to their differential structures and expression patterns, CCL19 and CCL21 display different binding affinities for specific heparin or heparan sulfate, and distinct signaling responses are required for *in vivo* functions (Rot and von Andrian, 2004; de Paz et al., 2007; Raju et al., 2015). CCR7 was the first identified lymphocyte-specific G-protein-coupled receptor (GPCR) with seven transmembrane spanning alpha helices (Birkenbach et al., 1993). CCR7 is expressed on double negative and single positive thymocytes, including naïve T cells, central memory T cells, regulatory T cells, naïve B cells, semi-mature/mature DCs and NK cells, and a minority of tumor cells, and it acts as a key regulator guiding homeostatic lymphocytes to secondary lymphoid organs (Ohl et al., 2004; Comerford et al., 2013; Hauser and Legler, 2016; Wang et al., 2018; Laufer et al., 2019). The CCR7-ligand axis carries out the following three fundamental “cellular reflexes”: message acquisition, semantic extraction and initiation of cell responses (Bardi et al., 2001; Rot and von Andrian, 2004; Griffith et al., 2014). Chemokine receptor internalization due to binding with a chemokine helps regulate chemokine activities (Rot and von Andrian, 2004). CCL19 is the only chemokine known to effectively stimulate β-arrestin-mediated CCR7 phosphorylation and internalization, leading to receptor desensitization and antigen-presenting dendritic cell (DC) migration (Bardi et al., 2001; Tian et al., 2014; Anderson C. et al., 2016). In particular, CCL19 displays obvious concentration- and time-dependent internalization in CD4^+^ and CD8^+^ T cells, which differs from CCL21 (Hjortø et al., 2016). Both ligands are able to activate G-protein signaling and elicit 3D chemotaxis and Ca^2+^ flux, but CCL19 has been shown to be relatively more potent (Bardi et al., 2001; Steen et al., 2014; Hjortø et al., 2016) (Figure 1).

Chemokines constitute a class of cytokines that control immunocyte migration to infection and inflammation sites in many biological processes. In different virus–host interactions, chemokine receptors may play a sensory function in the immune system, resulting in the production of the characteristic fingerprints of chemokines (Chensue, 2001; Alcami, 2003). The chemokine system can be mimicked by viruses, and viral proteins can act as antagonists or inappropriate agonists to use host chemokine receptors as modes of cellular invasion (Rot and von Andrian, 2004). For instance, human immunodeficiency virus type 1 (HIV-1) masquerades as a “chemokine” to promote its fusion with target cells (Murphy, 2001). Additionally, poxviruses and herpesviruses encode homologs of chemokine receptors that are expressed on their target cells, allowing the host chemokines to direct the infected cells to remote sites for viral dissemination (Alcami, 2003). Based on the essential roles of the CCL19-CCR7 axis in organizing immunological and inflammatory responses, we summarize in this review its pathogenic roles in some viral infection conditions, such as infections by HIV-1 (Wilflingseder et al., 2004; Cameron et al., 2010; Damås et al., 2012; Hong et al., 2012; Ramirez et al., 2014; Anderson J.L. et al., 2016), scrapie virus (Kim et al., 2018), respiratory syncytial virus (RSV) (Le Nouën et al., 2011; Inchley et al., 2013; Alturaiki et al., 2018), Epstein–Barr virus (EBV) (Ehlin-Henriksson et al., 2009; Dunham et al., 2017; Wu et al., 2017), influenza virus (Debes et al., 2004; Piqueras et al., 2006), dengue virus (DENV) (Wu et al., 2009, 2011; Hsu et al., 2015), hepatitis B virus (HBV) (Zhang et al., 2009; Cao et al., 2014), and West Nile virus (WNV) (Bardina et al., 2017). The CCL19-CCR7 axis also plays a role in vaccine-based protection against multiple viruses, such as HIV-1 (Hu et al., 2013), herpes simplex virus 1 (HSV-1) (Lee et al., 2003; Toka et al., 2003), HSV-2 (Yan et al., 2015), hepatitis C virus (HCV) (Hartoonian et al., 2014), and pseudorabies virus (Han et al., 2009). In addition, the CCL19-CCR7 interaction helps immune cells release antiviral-related cytokines (e.g., IFN-γ and IL-4), which promote T cell proliferation and antigen uptake by DC (Hu et al., 2013, 2017).

For many years, the focus on prophylactic vaccines aimed to elicit robust neutralizing antibody (Ab) responses. However, increasing evidence suggests that T cell-mediated immunity also plays a critical role in controlling persistent viral infections, such as HIV-1, cytomegalovirus (CMV), and HCV infections (Kallas et al., 2016). Recently, various promising prophylactic vaccines in conjunction with adjuvant cytokines or chemokines, such as CCL19, have been investigated to enhance virus-specific cellular immune responses through cytokine polyfunctionality. For future therapeutic initiatives, it is important to understand the roles of the CCL19/CCR7 axis in modulating immune cell migration and activation to potentially differentiate the good and bad effects. In this review, we evaluate the functional efficacies of CCL19 and CCR7 in viral infection and prevention, which may facilitate the development of more potent, durable and safe T cell-based anti-virus pharmaceuticals or vaccines.

## Decreased CCR7 Expression and Less Efficient Chemotactic Responses to CCL19 in the Context of Viral Infections

CCR7^+^ immune effector cells become dysfunctional during some viral infections, followed by the decreased expression of CCR7 during adaptive immune responses (Förster et al., 2008). CCR7 presents as a defining factor for non-polarized central (CCR7^+^) and polarized effector memory (CCR7^–^) T cells (Unsoeld et al., 2002). CCR7 is expressed at high levels on naïve and central memory T cells and enables homeostasis T cell subsets to recirculate and home to T cell areas in lymphoid organs, such as the white pulp areas of the spleen and lymph nodes (Rot and von Andrian, 2004; Schaerli and Moser, 2005). During murine lymphocytic choriomeningitis virus (LCMV) infection, CCR7 is down-regulated on virus-specific CD8^+^ effector T cells *in vivo* (Potsch et al., 1999). The down-regulation of CCR7 expression on virus-specific CD8^+^ effector T cells renders the cells unresponsive to chemokines from secondary lymphoid tissues, which limits T cell homing. It has been speculated that the exclusion of CD8^+^ effector T cells from the T-zone may represent an important mechanism protecting professional antigen-presenting cells (APCs) against cytotoxic T cell attacks and, thus, preventing a premature decline in immune responses. During HIV-1 infection, the CCL19/CCR7 axis assists with establishing a latent infection. The CCR7 expression pattern is strongly correlated with increased HIV-1 viral reservoirs and is associated with chronic HIV-1 infection. Stimulation of HIV-1-infected primary CD4^+^ T cells with CCL19 results in the enhancement of both the motility of CCR7-dependent T cells and the permissiveness of resting memory T cells, leading to the efficient propagation of HIV-1 (Saleh et al., 2007; Hayasaka et al., 2015). In addition, the HIV-1 accessory protein Vpu induces the down-regulation of CCR7 expression on the surface of HIV-1-infected CD4^+^ T cells (Ramirez et al., 2014). Consistently, a clinical study showed that HIV-1-infected individuals lack CCR7 expression on natural killer (NK) cells (Hong et al., 2012). An HIV-1 and CMV coinfection study demonstrated that most (>70%) CD8^+^ effector T cells have a CCR7^–^ phenotype in both the blood and lymph nodes (Ellefsen et al., 2002). Furthermore, mechanistic studies have shown that HIV-1 gp120 can mimic chemokine sequences and significantly promote chemokine receptor-dependent CD4^+^ target-cell migration to remote lymph nodes, which likely leads to enhanced viral dissemination (Murphy, 2001; Hayasaka et al., 2015).

Similarly, two previous studies shows that mice infected with scrapie virus (ME7 strain) have been shown to exhibit an impaired splenic white pulp structure and markedly diminished T-zone areas in the spleen due to the decreased splenic expression of CCL19 and CCL21 (Kim et al., 2016, 2018). Furthermore, decreased expression of T cell homing chemokines CCL19 and CCL21 resulted in a partial failure in CD4^+^ T cell recruitment to the spleen and expression of the memory marker CD44 on CCR7^±^ CD4^+^ T cells was decreased in scrapie virus (ME7 strain)-infected mice compared to that in control mice (Table 1). Finally, high levels of the cellular prion protein [PrP(C)] and accumulated PrP(Sc) expressed by follicular DCs were detected in the ME7-infected spleens. During the infection of respiratory viruses, such as human respiratory syncytial virus (HRSV), human metapneumovirus (HMPV) and human parainfluenza virus type 3 (HPIV3), can induce incomplete or short-lived virus-specific immunity. These viruses can produce symptomatic reinfections throughout life without undergoing significant antigenic changes. As a class of professional presentation cells, DCs can provide antigen presentation in the context of viral infection. However, human monocyte-derived DCs (mo-DCs) stimulated with HRSV, HMPV, or HPIV3 show an inefficient increase in CCR7 expression unless a secondary stimulation with lipopolysaccharide (LPS) or a cocktail of proinflammatory cytokines is presented. In contrast, HRSV and HMPV infections induce less CCR7 expression and less efficient DC migration in response to CCL19 than HPIV3. The low expression of CCR7 mediates the inefficient migration of HRSV- and HMPV-stimulated DCs to lymphoid organs and causes impaired adaptive responses to these viruses, ultimately leading to abundant virus replication and tissue damage, thus resulting in more severe disease (Le Nouën et al., 2011; Inchley et al., 2013) (Table 1).

**TABLE 1:** Expression of CCL19 and its receptor CCR7 during viral infections.

**Virus**	**Species**	**Early stage**	**Middle stage**	**End stage**	**Outcome**	**References**
HIV-1	Human	–	–	CCR7^+^ CD45RA^+^ CD4^+^ T cells ↓	Chronic infection/latency	Hong et al., 2012; Ramirez et al., 2014
Scrapie virus	Mice	Days 1–50: CCR7^+^ CD4^+^ T cells were normal	Days 51–130: CCR7^+^ CD4^+^ T cells were normal	Days 131–200: CCR7^+^ CD4^+^ T cells ↓	Diminished T-zone area in the spleen and increased germinal center reactions.	Kim et al., 2016, 2018
RSV	Human	–	–	CCR7^+^ mo-DCs ↓	Less DCs migrate to lymphatic tissue	Le Nouën et al., 2011; Inchley et al., 2013
	Mouse DCs	Day 1: CCL13↑, CCL12, CCL19, and CCL21	Day 2: CCL13↑, CCL12, CCL19, and CCL21	Days 4 and 7: CCL12, CCL13, CCL19↑, and CCL21	Unknown	Alturaiki et al., 2018
EBV	Tonsillar B cells	Day 2: CCR7↓, CXCR5↓, CCR9↑, CCR2↑, CCL19↑ (2.9-fold), and CCL20↑ (4.1-fold)	Day 7: CCR7↓, CXCR5↓, CCR6↓, CCL2↓, CXCL2↓, CCL11↑, CCL24↑, CCL1↑, and CCL13↑	Day 14: CCR7^–^ and CXCR5^–^	CCL19-induced migration may be impaired even in the presence of CCR7.	Ehlin-Henriksson et al., 2009
DENV	human	–	–	Hour 48: CCR7^+^ mo-DCs↑	Help DENV infect mo-DCs	Wu et al., 2009; Hsu et al., 2015
Influenza A virus	Mice	–	–	Day 8: CCR7↓ and α4β7↓ in the lung parenchyma and CCR7↑ in the lung draining lymph node	T cells at different anatomical sites represent the most differentiated effector cell type and lack the ability to recirculate.	Debes et al., 2004
	Human	Hours 2–4: CXCL16, CXCL1, CXCL2, and CXCL3↑	Hours 8–12: CXCL8, CCL3, CCL4, CCL5, CXCL9, CXCL10, and CXCL11↑	Hours 24–48: CCL19, CCL22, and CXCL13↑	Attract naïve T and B lymphocytes	Piqueras et al., 2006
HBV	Mice	–	–	CCR7^+^, CD45RA^+^, CD127^+^, and CD8^+^ T cells↑	Help chronic infection	Zhang et al., 2009; Cao et al., 2014
WNV	Mice	–	–	CCR7^+^ mDCs↑	Absence of CCR7 results in the dysregulation of the number of circulating T cells; CCR7-deficient mice have a defect in CNS viral clearance; CCR7 is a gatekeeper for non-specific viral transference to the brain.	Bardina et al., 2017

During EBV infection, CCR7 expression likely plays an important role in pathogenesis. Compared with uninfected tonsillar B cells, CCR7 expression, which is critical for the migration of B cells into lymphoid tissues, has been shown to decline from day 2 after EBV infection and be undetectable by day 14 (Ehlin-Henriksson et al., 2009) (Table 1). In addition, migration of EBV-infected cells toward CCL19 or CCL21 is impaired, although the expression of CCR7 in infected cells is similar to that in uninfected controls (Ehlin-Henriksson et al., 2009; Hauser and Legler, 2016; Dunham et al., 2017; Wu et al., 2017). CCR7 plays an essential role in B cell trafficking in lymphoid tissue, and its activation has been shown to be strictly dependent on a highly conserved cellular DNA binding factor (CBF1) as a blockade of or a deficiency in this factor results in the repression of immunoglobulin (Ig) expression in the context of EBV infection (Maier et al., 2005, 2006).

**FIGURE 2:**
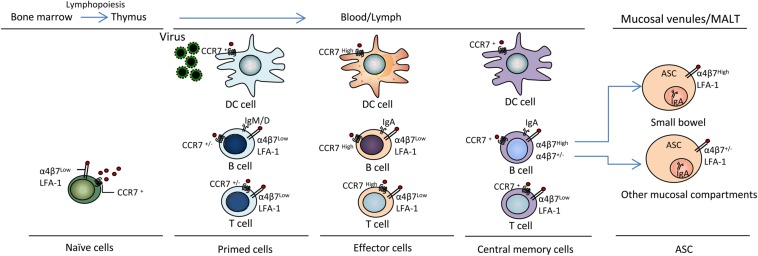
CCL19 and CCR7 mediate virus antigen uptake and immunocyte activation and differentiation. DC, B and T lymphopoiesis are characterized by sequential changes in migration properties. Surface receptor and Ig expression occurs during T lymphopoiesis in bone marrow and the thymus during immune response initiation and the generation of effector T cells for participation in active defense at virus entry sites in peripheral tissues, including blood, lymph, mucosal venules and MALT; thereby, inflammation and the establishment of memory cells for immune surveillance occur. α4β7, integrin α4β7; LFA-1, lymphocyte function-associated antigen-1; ASC, antibody-secreting cell; MALT, mucosal-associated lymphoid tissue. Other mucosal compartments: colon, alveolus, mammary and salivary glands, genital mucosa.

During Influenza A virus infection, the Th1-based immune response dominates the immune process (Doherty et al., 1997). CCL19/CCL21 and CCR7 are well-known to be essential for fulfilling the important role of recruiting T cells into the lung and other peripheral specialized microenvironments within tissues (important pathogen entry sites). CCR7 plays an important role in the migration of T cells from lymph nodes and Peyer’s patches through high endothelial venules during mucosal immune protection (Förster et al., 1999; Debes et al., 2004). However, compared to uninfected mice, CCR7 expression has been shown to be down-regulated on influenza virus-specific CD4^+^ T cells obtained from the spleen, mesenteric lymph nodes (MLNs) and the lungs at the peak of infection (Debes et al., 2004) (Figure 2), indicating that these cells lost the ability to recirculate after the response to viral antigens (Ags).

## Increased CCR7 Expression in the Context of Denv Infections

High expression of CCR7 on immunocytes has been observed in DENV infection. DCs are the most important target cells, and CCR7 expression has been shown to be increased on DENV-stimulated mo-DCs (Wu et al., 2011). However, mo-DCs with increased CCR7 expression have a greater opportunity to interact with abundant T cells and begin to secrete cytokines, metalloproteinases, and chemokines in the T-zone of draining lymph nodes. The clinical manifestations of DENV infection include dengue fever and potentially fatal phenomena, such as dengue hemorrhagic fever (DHF) and dengue shock syndrome (DSS). The interaction between DENV and target immune effectors, cytokines or chemokines may lead to the development of severe clinical manifestations, such as DHF (Wu et al., 2009). In clinical therapy, some medicines, such as leflunomide, inhibit the excessive production of cytokines and chemokines from DENV-infected mo-DCs by suppressing mo-DC maturation (Wu et al., 2011). Similarly, DENV infection has been shown to specifically increase the mRNA and protein levels of Gal-9. Additionally, Gal-9 small interfering RNA downregulates CCR7 expression and suppresses DENV-specific DC migration toward the chemoattractants CCL19 and CCL21. Thus, a Gal-9 inhibitor might be useful for preventing immunopathogenesis in DENV infection (Hsu et al., 2015). Similar functional molecules might be useful in drug development to prevent DENV immunopathogenesis by reducing the number of CCR7-expressing cells.

## CCL19 Helps Establish Viral Integration and Latency and Down-Regulates CCR7 Expression in HIV-1 Infection

Via interacting with its receptor (CCR7), CCL19 has the potential to activate the signaling pathway enabling HIV-1 to enter the nucleus of resting (memory) T cells (Saleh et al., 2016; Cameron et al., 2010; Anderson J.L. et al., 2016). HIV-1 latent infection in resting memory CD4^+^ T cells is a major barrier to HIV-1 eradication. The reversal of proviral latency has attracted much attention as a curative strategy for HIV-1 infection (Cillo et al., 2014). CCL19-treated CXCR4-expressing CD4^+^ T cells have been shown to exhibit increased permissiveness for HIV-1 production, thereby facilitating provirus post-integration and latency (Anderson J.L. et al., 2016). Furthermore, inoculation of CD4^+^ T cells with HIV causes a modest down-regulation (as shown via flow cytometry) of CCR7 expression and a slight increase of CCR5 expression after stimulation with CCL19 (Anderson J.L. et al., 2016). CCR5 is a co-receptor essential for HIV-1 entry into susceptible cells and is an attractive target for controlling HIV-1 infection (Li et al., 2015). In addition, CCL19 and mDCs co-culture with CD4^+^ T cells has been shown to be beneficial for CCR5- and CXCR4-tropic virus latent infection *in vitro* (Anderson J.L. et al., 2016).

## CCR7 Is Required for the Sufficient Migration of Mature Dendritic Cells (mDCs) and T Cells Into the Draining Lymph Nodes Following Viral Infections

In the context of chronic HBV infection, mDCs are highly migratory, which is accompanied by the up-regulation of CCR7 expression on hepatic mDCs and an increase in the response to CCL19 (Abe et al., 2004). In two studies, immature hepatic DCs did not respond to any tested chemokines, despite the expression of mRNA transcripts encoding the appropriate receptors for these chemokines, and CCR7 expression was strongly enhanced in response to DC maturation (Abe et al., 2004; Thomson and Knolle, 2010). Hepatic mDCs play a critical role in promoting immune tolerance by producing IL-10 and TGFβ, activating regulatory T cells or regulatory B cells and suppressing effector T cell proliferation (Abe et al., 2004; Liu et al., 2018). Compared with the robust responses at clinical onset, HBV-specific CD8^+^ T cell responses are rather weak and limited, which may lead to viral persistence and disease progression (Nitschke et al., 2016; Fisicaro et al., 2017; Wieland et al., 2017). However, acute inflammation may convert hepatic mDCs from a tolerogenic phenotype, allowing these cells to activate T cells (Abe et al., 2004; Thomson and Knolle, 2010).

CCR7 expression can induce the migration of antigen-specific effector and central memory T cells to the lymph nodes via the chemotactic response to CCL19 (Abe et al., 2004). The major phenotype and functions of pathogen-specific CD8^+^ T cells may differ in different viral infections (Appay et al., 2002; Romero et al., 2007). The quantitative and qualitative compositions of the immune cells in the liver markedly differ from those in secondary lymphoid organs, including the spleen, lymph nodes and peripheral blood. The hepatic CD8^+^/CD4^+^ T cell ratio is 3.5:1; however, this ratio is the reverse of the 1:2 CD8^+^/CD4^+^ T cell ratio found in secondary lymphoid organs (Horst et al., 2016). CD8^+^ T cells play crucial roles in HBV control and liver inflammation. Several studies have investigated CCR7 expression by HBV-specific CD8^+^ T cells in the context of chronic HBV infection (Boettler et al., 2006; Zhang et al., 2009). Up-regulation of programed death-1 (PD-1) expression can impair HBV-specific memory CD8^+^ T cell responses, resulting in the functional suppression of IFN-γ production. Additionally, blockage of PD-1/PD-L1 interactions *in vitro* increases the frequency of HBV-specific CD8^+^ T cells, and enhances CCR7, CD45RA, and CD127 expression in these cells, resulting in increased cell proliferation and IFN-γ production (Zhang et al., 2009) (Table 1).

Chronic infections with blood-borne pathogens, such as HIV-1, HBV and HCV, tend to increase the development of memory homeostasis T cells (Carotenuto et al., 2011; Cao et al., 2014; Cillo et al., 2014; Baskic et al., 2017). During primary viral infections, virus-specific T cell responses are vigorous; however, once a persistent infection is established, both virus-specific CD4^+^ and CD8^+^ T cells become dysfunctional or difficult to detect *ex vivo* (Cao et al., 2014). Virus-specific CD8^+^ effector T cells play a critical role in eliminating HIV-1, EBV, HBV, CMV and HCV infections, and the expression patterns of CCR7, CD27, and CD28 exhibit similar characteristics during the primary infection phase and chronic phase of a persistent infection (Appay et al., 2002; Romero et al., 2007). The CCR7^+^ CD27^+^, CCR7^–^ CD27^+^, and CCR7^–^ CD27^–^ phenotypes can represent the early, median, and late stages of memory CD8^+^ T cell differentiation, respectively, while a down-regulated expression of these molecules indicates an inability of T cells to differentiate toward the effector phenotype. Several studies report that the removal of effector T cells from circulation favors the recruitment of CCR7^+^ naïve cells, which may result in an impairment in the generation of functionally competent memory T cells and an inability to control viral replication (Appay et al., 2002).

Mature dendritic cells and T cells that express CCR7 enhance the velocity of T cell locomotion within the lymph nodes and, thus, increase the likelihood that T cells encounter DCs (Mora and von Andrian, 2008). In influenza virus infection, high levels of CCR7^+^ IFN-γ^+^ CD4^+^ T cells have been observed in lung draining lymph nodes (Debes et al., 2004). A study investigating the kinetics of chemokine expression showed that the levels of CCL19, CCL22, and CXCL13 are significantly up-regulated during the third wave (8 to 24 h) of influenza virus infection, which helps CCR7^+^ DCs reach the lymphoid organs and attract naïve T and B cells (Piqueras et al., 2006) (Table 1).

The major adaptive immune lymphocytes present in the lungs of human infants who have died from severe RSV infection are B cells (Welliver et al., 2007; Reed et al., 2009). The B cell differentiation factor BAFF is an indicator of pulmonary Ab responses after HRSV, HMPV, H1N1, bocavirus, rhinovirus and *Mycoplasma pneumoniae* infections (McNamara et al., 2013). CCL19 and other chemokines, particularly CXCL12, CXCL13 and CCL21, influence B cell and human blood DC (i.e., plasmacytoid and myeloid DCs) differentiation, migration and homeostasis (Freire-de-Lima et al., 2017; Alturaiki et al., 2018). The expression kinetics of these chemokines has been reported to be related to the airway epithelial innate immune response to respiratory virus infections. CCL19 has been shown to be expressed in lung tissues 1, 2, and 7 days after infection with RSV (Le Nouën et al., 2011; Inchley et al., 2013; Alturaiki et al., 2018). However, in the context of influenza virus infection, CCL19 and CCL21 levels have been reported to be increased during the late wave (Comerford et al., 2006). These variable results may be explained by differences in the models or sampling methods used (Alturaiki et al., 2018) (Table 1).

During homeostasis, CCR7 regulates the homing of T cells into lymphoid organs. During WNV infections, CCR7^+^ DCs regulate the homing of T cells expressing the cognate ligands CCL19 or CCL21 into the lymph nodes immediately following infection and restrict leukocyte migration into the brain. Leukocyte hypercellularity within the central nervous system (CNS) contributes to CNS viremia, neuroinflammation, and increased mortality. Thus, CCR7 acts as a host defense restriction factor limiting neuroinflammation during acute WNV infection (Bardina et al., 2017) (Table 1).

Taken together, these data show that host responses to viral infections involve distinct effectors of innate and adaptive immunity and that the lymphocyte mobilization mediated by these effectors needs to be coordinated to ensure protection. Both inflammatory and homeostatic chemokines are involved in the control of the trafficking of effector or memory cells. Inflammatory chemokines determine the cellular infiltrates at sites of pathogen entry, whereas inflammation and homeostatic chemokines regulate the inflammation-independent, continuous trafficking of memory cells through healthy peripheral tissues.

## CCL19-CCR7 Axis Signaling Pathways in Viral Infections

**FIGURE 3:**
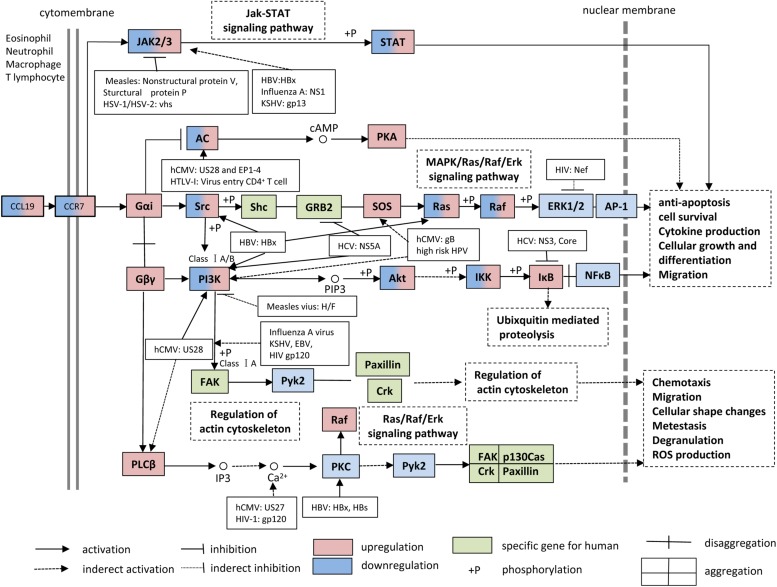
CCL19 and CCR7 signaling pathways. During viral infections, many studies have supported the notion that the interaction between CCL19 and CCR7 facilitates the up-regulation/down-regulation of downstream signaling adaptor molecules and results in anti-apoptosis, cell survival, cytokine production, cellular growth, differentiation, chemotaxis, and migration, etc. KSHV, Kaposi sarcoma-associated herpesvirus; HTLV, human T-cell leukemia virus 1; HPV, human papillomavirus.

The CCL19-CCR7 axis is involved in the constitutive migration and homing of lymphocytes. Furthermore, the CCL19-CCR7 axis has been shown to perform other biological activities, such as regulation of DC morphologic change and thymic T cell development and suppression of DC apoptosis, which can lead to the regulation of adaptive immunity and tolerance (Yanagawa and Onoe, 2002; Müller and Lipp, 2003; Sánchez-Sánchez et al., 2004; Förster et al., 2008; Raju et al., 2015). This axis performs its biological functions by activation of the G protein-coupled receptor kinase (GPK)/β-arrestin that transduces the binding of extracellular stimuli to intracellular signaling (Zidar et al., 2009; Tian et al., 2014). CCL19 but not CCL21 induces robust β-arrestin 2 recruitment and results in serine/threonine-phosphorylated receptor CCR7 internalization to endosomal vesicles, thereby efficiently limits receptor susceptibility to extracellular ligands (Kohout et al., 2004) (Figure 1). In addition, CCL19 has been shown to be more efficient than CCL21 in activating ERK1/2 (part of the MAP kinase cascade) through Gαi subunit (Kohout et al., 2004; Raju et al., 2015) and increasing Ca^2+^ flux through the Gβγ subunit (Yoshida et al., 1998; Otero et al., 2008) (Figure 3). CCR7, as a homeostatic chemokine receptor, inhibits adenylate cyclase, and limits the level of intracellular cyclic adenosine monophosphate (cAMP) and the activation of protein kinase A (PKA) (Steen et al., 2014; Raju et al., 2015). Similar to other GPCRs, CCR7 plays a crucial role in activating or inhibiting downstream signaling adaptors in viral infections through G-protein-promoted secondary messengers, including cAMP, Ca^2+^, and phosphoinositides (Steen et al., 2014; Raju et al., 2015) (Figure 3). The following three principle modes are involved in the GPCR homeostatic regulation: desensitization (receptor becomes refractory to continued stimuli), internalization (receptors are physically removed from the cell surface by endocytosis) and down-regulation (cellular receptor levels are decreased) (Tian et al., 2014). In addition to inducing directional steering in cells, CCR7 provides costimulatory and survival cues (Bock et al., 2016). Moreover, CCR7-mediated human T cell polarization and migration have been shown to be linked to protein–protein interactions in cell signaling across multiple cellular compartments (Raju et al., 2015).

Over the past few years, numerous CCL19-based signaling-related adaptor molecules have been reported in CCR7 signaling pathways, and their specific functions during viral infections are summarized in Figure 3. Viruses have been well-demonstrated to have highly efficient strategies to modulate and prevent the transduction of apoptotic signals to favor their infections through viral proteins, such as the HBx gene product of HBV (Shin et al., 2016). For this review, we searched CCR7-associated chemokine signaling pathways in the KEGG PATHWAY Database^1^ and discuss the multiple regulatory mechanisms of CCR7 signaling and the influences on CCR7 functions during viral infections.

The HIV-1 accessory protein Vpu reduces CCR7 expression on CD4^+^ T cells (Ramirez et al., 2014). Vpu specifically interacts and colocalizes with CCR7 in the *trans*-Golgi network in which CCR7 is retained. The stimulation of HIV-1-infected primary CD4^+^ T cells with CCL19 reduces mobilization of Ca^2+^, reduces phosphorylation of Erk1/2, and impairs migration toward CCL19 (Ramirez et al., 2014). Studies concerning CCL19-CCR7 signaling have shown that CCL19-induced signaling proteins mediate HIV-1 integration in CD4^+^ T cells at several integration sites and that this process is suppressed by inhibitors of the PI3K, NF-κB and MEK/Ras/Raf signaling pathways (Raju et al., 2015; Saleh et al., 2016) (Figure 3). Recently, the Food and Drug Administration (FDA) approved the Janus Kinase (JAK) inhibitor ruxolitinib to diminish the release of multiple cytokines and thereby prevent their effects on latency reversal in HIV-1-positive patients. A combination of ingenol compounds with the JAK inhibitor may represent a novel strategy for HIV-1 eradication (Spivak et al., 2016).

The infection of DCs with DENV causes cell maturation and probably enhances cell migration to lymphoid organs to promote interactions with T cells (Mathew and Rothman, 2008). At 48 h post-infection, the number of CCR7^+^ mo-DCs increases, and the increase in cell number is accompanied by the significant activation of the COX-2-PGE_2_ signaling pathway in migrating DCs (Wu et al., 2009). All MAPK inhibitors and the COX-2 inhibitor celecoxib suppress DENV-induced PGE_2_ production to basal levels. The mechanisms involved in the activation of COX-2 include the activation of the IKK-NF-κB and MAPK-activator protein-1 (AP-1) signaling pathways (Wu et al., 2009; Hsu et al., 2015). Several COX-2 upstream signaling molecules have been suggested to be useful for the treatment or control of viral infections. For example, the blockade of ERK, which is upstream of the MKK1/2 kinase, suppresses virus infectivity by inhibiting early gene expression in human CMV (hCMV) infections (Johnson et al., 2001). Additionally, leflunomide has been shown to be an effective therapeutic drug for DENV infection. Leflunomide inhibits DENV-induced mo-DC migration in response to the chemoattractant CCL19 by suppressing CCR7 expression and the NF-κB and AP-1 signaling pathways (Wu et al., 2011).

## CCL19 as a Molecular Adjuvant Enhances Virus-Specific Immune Responses

Cytokines have been successfully used as molecular adjuvants to promote virus-specific humoral and cellular immune responses by modifying the magnitude, intensity, nature, and duration of the responses (Sin et al., 1999; Kanagavelu et al., 2012; Gupta et al., 2015; Doosti et al., 2019). The positive effects of chemokines as immunomodulators of vaccines against viral infections remain to be further evaluated. CCL19 has been postulated as a promising adjuvant candidate for vaccines against both cancers and infectious diseases due to its paramount importance in immune response formation and its diverse effects on DC and lymphocyte migration and activation. CCL19 also controls lymphocyte localization during T cell development in response to immunization. Activated T cells lead to alterations in the expression of various molecules, including integrins (α_4_β_3__,_ LFA-1), selectins, and chemokine receptors, leading to the modulation of key intracellular signaling events that promote T cell proliferation, differentiation, and migration to inflamed tissues (Murdoch and Finn, 2000; Tahamtan et al., 2018; Justo-Junior et al., 2019) (Figure 2).

In the context of viral neutralizing antibodies (nAbs), studies have shown that both monomeric gp120 and trimeric gp140 HIV-1 vaccines induce neutralizing antibody responses but still lack sufficient breadth to effectively protect against diverse HIV-1 isolates (García-Arriaza et al., 2017; Shen et al., 2017). HSV-2 gB or gD subunit vaccines could induce high levels of neutralizing Abs but could not efficiently protect mice against a viral challenge (Zhu et al., 2014). Thus, effective vaccines may be needed to broadly induce neutralizing Abs. DNA vaccines are generally considered suboptimal for inducing humoral responses, particularly in humans. Strategies to overcome these limitations include the optimization of plasmid backbones, the use of molecular adjuvants and changing the delivery methods. We previously explored the possibility that the use of chemokine CCL19 or CCL28 could optimize systemic and mucosal humoral responses to a HIV-1 vaccine candidate gp140, which is the primary target of Ab-mediated antiviral functions (Hu et al., 2013). In the CCL19 adjuvant group, CCR7 showed a high expression on activated B cells, T cells and DCs in secondary lymphoid organs, which was beneficial for increasing the chance of interactions among B cells, T cells, and DCs in the local tissue (Table 2).

**TABLE 2:** CCL19-based adjuvant in virus vaccines.

**Virus**	**Approaches**	**Antigens**	**Humoral immunity**	**Cellular immunity**	**Virulent challenge**	**Chemotaxis of secondary lymphoid cells**	**Mechanisms**	**Reference**
HIV-1	i.m., i.n.	gp140	Total IgG, IgG1, IgG2a IgG1 > IgG2a, IgA	Th1, Th2	–	DCs, T cells, IgA ASCs	Mobilize CCR7^+^ DCs, T cells and IgA ASCs into secondary lymph nodes and mucosal tissues.	Hu et al., 2013
HSV-1	i.n.	gB	IgG, IgA	CTL	Survival	DCs, memory CD8^+^ T cells	CCL19 induces protective DCs and memory CD8^+^ T cell responses, which generate IFN-γ against HSV-1.	Lee et al., 2003; Toka et al., 2003
HSV-2	i.m.	gB	Total IgG, IgG2a, IgG2b, IgG3, IgA	Th1, Th2	Survival	DCs, T cells, IgA ASCs	CCL19 recruits responsive T cells, DCs, and IgA ASCs to secondary lymph nodes and mucosal tissues.	Yan et al., 2015
HCV	i.m.	Core DNA	Total IgG, IgG2a	Th1	–	–	Facilitates the chance of interaction among DC, T and B cells in lymphoid tissues and consequently enhances both humoral and cell-mediated immune responses.	Hartoonian et al., 2014
Pseudorabies virus	Genetic co-transfer	gB	IgG2a	Th1	Survival	DCs	Increased encounter frequency between APCs and T cells may explain the enhanced immune response observed after the genetic co-transfer of CCR7 ligands with the PrV DNA vaccine.	Han et al., 2009

In the context of cellular immune responses, several potential roles of molecular adjuvants, such as cytokines and costimulatory molecules, in vaccination strategies have been investigated. Currently, various promising prophylactic vaccines focused on inducing substantial vaccine-specific T cell responses have been developed (Hartoonian et al., 2014; Rosendahl Huber et al., 2014). Increased breadth in the vaccine-induced T cell response has been found to be beneficial against many chronic pathogens (Kallas et al., 2016; Panagioti et al., 2016). The molecular adjuvant CCL19 plays an important role in augmenting the trafficking of T cell-based vaccines into regional T cell compartments through efficient CCR7 gene transduction and participates in the priming and preparation of antigen-specific T cells and the production of Abs (Müller and Lipp, 2003; Aritomi et al., 2010).

In chronic viral infections, memory B and T cells are more numerically and functionally superior to neutralizing antibodies than the corresponding naïve precursor cells that are present before infection as memory lymphocytes tend to induce rapid and powerful recall responses (Panagioti et al., 2016, 2018). Because neutralizing antibodies fail to provide control over persisting viral infections (e.g., herpesviruses, HIV-1, and HCV), therapeutic immunizations should focus on generating strong cellular T cell-based immunity and more virus-specific memory immunocytes. Live attenuated, synthetic and subunit vaccines are all able to elicit central memory T cells, effector memory T cells and resident memory T cells, and these responses are highly dependent on the addition of adjuvants and the route of administration (Schaerli and Moser, 2005; Mueller et al., 2013; Khan et al., 2017; Gebhardt et al., 2018).

Substantial evidence indicates that CCL19-adjuvanted vaccine recipients produce CD4^+^/CD8^+^ T cells more promptly and at higher levels than recipients administered a control vector lacking CCL19 (Table 2). In a study coadministering HSV-1 vaccine and plasmids encoding CCL19 (pCCL19) and HSV-1 gB (pgB), CCR7^high^ DCs were activated and migrated, resulting in the enhancement of antigen uptake and presumably the augmentation of naïve T cell priming. Compared with pCCL19 delivered alone, the codelivery of pCCL19 and pgB induced a notable increase in lung CD11c^+^ DCs. In the pCCL19-codelivery group, the CD8^+^ T cells isolated from the lungs produced more IFN-γ. Consistently, it appears that CCL19 codelivery may lead to the induction of functional memory CD8^+^ T cells (Lee et al., 2003; Toka et al., 2003). The high level of IFN-γ observed in these CD8^+^ T cells may suggest that the enhanced potential to secrete IFN-γ plays a notable protective role in lethal mucosal HSV-1 challenges. In our HIV-1 and HSV-2 vaccine studies, adjuvant CCL19 codelivery produces a balanced enhancement of HIV-1 gp140- or HSV-2 gB-specific Th1/Th2 responses (Hu et al., 2013; Yan et al., 2015). Such enhancements appear to be associated with the mobilization of abundant CCR7^+^ immunocyte migration to secondary lymphoid organs and mucosal tissue. Similarly, a previous HCV vaccine study showed that CCL19 contributes to the activation of DCs and CD4^+^ T cells, enhances CD8^+^ T cell accumulation and helps attract rare virus-specific T cells in the context of HCV core DNA vaccination in mice (Hartoonian et al., 2014). CCL19 as molecular adjuvant is helpful in increasing the naïve T cell sensitivity to low density Ag presentation. Such functional studies might be helpful for human virus vaccine development.

Regarding virus challenges, whether enhanced immunity mediated by adjuvant CCL19 can protect animals against genital tract mucosal infections has been tested, and previous studies have demonstrated that CCL19 enhances the protective responses to an HSV-1 challenge (Lee et al., 2003; Toka et al., 2003). In these studies, a CCL19 protective vaccination regimen was shown to elicit increased serum and vaginal HSV-1 gB-specific IgG and IgA Ab levels through intranasal mucosal immunization. CCL19 appeared to play a pivotal role in increasing Ab responses (levels of total Abs, virus-specific Abs and neutralizing Abs). Furthermore, CCL19 enhanced gB-specific immune responses by improving the kinetics and distribution of the adaptive immune response to the codelivered antigen and protected animals against genital infection by HSV-2. Finally, the results showed that the fusion constructs induced strong HSV-2 gB-specific IgG and IgA Ab levels in mouse sera and vaginal fluids. In a different study, we revealed that a gB-CCL19 fusion construct exhibited a stronger ability to increase the numbers of CCR7^+^ immunocytes in secondary lymphoid tissue and IgA^+^ cells in mucosal sites (Figure 2). The enhanced systemic and vaginal mucosal Abs protected the tested mice against a lethal intravaginal HSV-2 challenge *in vivo* (Yan et al., 2015). These findings may provide an effective strategy for the design of vaccines against mucosal infection by sexually transmitted viruses.

## Conclusion and Perspectives for CCL19 Use for the Prevention of Viral Infections

This review seeks to address how we can apply the current understanding of CCL19 and CCR7 functions to better understand the pathogenesis of viral infections and better treat or prevent viral diseases. CCL19 and CCR7 are essential players in the balance between immunity and tolerance. CCL19 promotes activation-induced cell death (AICD) in antigen-specific CD4^+^ T cells, and this chemokine regulates not only T cell mobilization but also the post-activation fate of T cells (Yasuda et al., 2007; Förster et al., 2008). It is a key step to understanding the appropriate initial programing signals as the signaling pathways of CCR7 elicited by CCL19 during priming or boosting influences the development of lymphocytes. Information regarding the differences in anatomical location, activation, and differentiation between memory T cells in lymphoid organs and those in non-inducing lymphoid organs could also be very valuable (Thomson and Knolle, 2010). We believe that recent advances in the field of target cell signaling stimulation coupled with animal models that express viral antigens could provide an opportunity to directly address some of these questions in a living animal. Such work will not only improve our understanding of CCL19 and CCR7 expression and their functions during viral infections but may also provide tools for the design of new therapeutic strategies for the treatment of viral infections and the prevention of invading pathogens.

Several lymphocyte-targeted chemokines, such as CCL19, CCL21 and CCL28, are known to be important for regulating adaptive immune responses during viral infection, and their engagement with cognate receptors can result in enhanced T cell activation, expansion, and survival as well as the establishment of long-term memory. The most important feature of the immune system is its ability to produce protective immune responses against pathogens while maintaining tolerance to self-antigens and innocuous environmental Ags. Therefore, chemokines have the potential to serve as effective immunomodulatory components of prophylactic vaccines against chronic viruses (Hartoonian et al., 2014; Kallas et al., 2016).

## Author Contributions

YY, RC, XW, and KH analyzed the data. YY drafted the manuscript. LH, ML, and QH reviewed and finalized the manuscript. All authors read and approved the final manuscript.

## Conflict of Interest

The authors declare that the research was conducted in the absence of any commercial or financial relationships that could be construed as a potential conflict of interest.

## Footnotes

1 https://www.kegg.jp/kegg-bin/show_pathway?ko04062+K04182
